# Gene Regulatory Network Guided Investigations and Engineering of Storage Root Development in Root Crops

**DOI:** 10.3389/fpls.2020.00762

**Published:** 2020-06-17

**Authors:** Nam V. Hoang, Chulmin Park, Muhammad Kamran, Ji-Young Lee

**Affiliations:** ^1^School of Biological Sciences, Seoul National University, Seoul, South Korea; ^2^Plant Genomics and Breeding Institute, Seoul National University, Seoul, South Korea

**Keywords:** gene regulatory network, GRN, storage root development, secondary growth, cambium, stress-responsive gene, root crop yield engineering

## Abstract

The plasticity of plant development relies on its ability to balance growth and stress resistance. To do this, plants have established highly coordinated gene regulatory networks (GRNs) of the transcription factors and signaling components involved in developmental processes and stress responses. In root crops, yields of storage roots are mainly determined by secondary growth driven by the vascular cambium. In relation to this, a dynamic yet intricate GRN should operate in the vascular cambium, in coordination with environmental changes. Despite the significance of root crops as food sources, GRNs wired to mediate secondary growth in the storage root have just begun to emerge, specifically with the study of the radish. Gene expression data available with regard to other important root crops are not detailed enough for us directly to infer underlying molecular mechanisms. Thus, in this review, we provide a general overview of the regulatory programs governing the development and functions of the vascular cambium in model systems, and the role of the vascular cambium on the growth and yield potential of the storage roots in root crops. We then undertake a reanalysis of recent gene expression data generated for major root crops and discuss common GRNs involved in the vascular cambium-driven secondary growth in storage roots using the wealth of information available in Arabidopsis. Finally, we propose future engineering schemes for improving root crop yields by modifying potential key nodes in GRNs.

## Introduction

In natural conditions, plants are exposed simultaneously to several sources of abiotic and biotic stresses, which could negatively influence their growth and vitality (Pandey et al., 2017; Schmidt and van Dongen, 2019). To cope with these stresses, plants have evolved to allocate their resources resiliently, achieving a balance between growth processes and stress resistance (Vermeirssen et al., 2014; Miao et al., 2015; Luo et al., 2016; Van den Broeck et al., 2017). Such evolutionary processes have accompanied the establishment of highly coordinated gene regulatory networks (GRNs) of developmental regulators and/or stress-responsive genes (Miller et al., 2015; Van den Broeck et al., 2017). Theoretically, this involves interactions between thousands of genes in the plant genome and several environmental factors of different complexity levels (Marshall-Colon and Kliebenstein, 2019).

It is widely accepted that plant growth has an antagonistic relationship with stress resistance, as a trade-off exists between them. At the molecular level, this corresponds to the induction of stress-responsive gene cascades and the suppression of growth-promoting regulators, allowing the plants to prioritize their defense responses for survival (Huot et al., 2014; Kudo et al., 2019). Interesting enough, there is accumulating evidence that suggests a positive (or dual) role of many stress-responsive genes in plant development (Wang J. et al., 2018; Wang P. et al., 2018). Currently, the underlying molecular mechanism is not well understood, and our understanding is limited to mostly controlled environments and small biological scales. It would be critical to uncover the details of how stress-responsive genes coordinate with developmental regulators and to determine why the antagonism is strong in some cases, but not in others. This would aid in the identification of potential targets for genetic modification to sustain and improve crop productivity in ever-changing environments through the breeding of stress-tolerant crops.

The molecular mechanisms of several GRNs in regulating plant developmental processes and plant responses to environmental stimuli have been investigated in both model plants and several other plant systems. These GRN studies cover important developmental processes ranging from the circadian clock (Harmer et al., 2000), flowering (Nagel and Kay, 2012; Luo et al., 2016) to cell specification and secondary cell-wall biosynthesis (Taylor-Teeples et al., 2015; Chen et al., 2019). GRNs involved in plant responses to stresses were also elucidated; examples include transcription factor (TF) networks in response to abiotic stresses (Vermeirssen et al., 2014; Miao et al., 2015; Van den Broeck et al., 2017) and biotic stresses [reviewed by Tsuda and Somssich (2015)]. Together, these GRNs serve as a good foundation for understanding how genes interact to maintain a molecular balance, as well as the dynamic regulation when there are external stresses that disrupt the aforementioned growth-stress response balance. By incorporating large-scale time-course and tissue-specific data obtained from high-throughput platforms in recent years, these networks provide additional enriched and detailed information regarding their spatial and temporal regulation.

Root crops (also known as root tubers, e.g., cassava, sweet potato, carrot, and radish), together with tuber crops (also known as stem tubers, e.g., potato), make up an essential part of the diet and food industry, being globally important sources of carbohydrates (only after cereals), sugar, and vegetables (Hahn, 1977; Asadi, 2006; Jansson et al., 2009; Villordon et al., 2014; Nishio, 2017; Jiang et al., 2019; Simon, 2019). These crops are particularly important food sources in environmentally harsh areas. In root crops, storage roots result from the thickening of primary or adventitious roots. This root thickening, also referred to as secondary growth or radial growth, is mainly driven by the vascular cambium, one of the secondary meristems established post-embryonically (Zhang et al., 2011; Tonn and Greb, 2017). The vascular cambium is established via the division and reorganization of cells derived from the procambium and its neighboring cells: pericycles in roots and the parenchyma cells between vascular bundles in stems, then giving rise to cells constituting the xylem inward and phloem outward (i.e., secondary xylem and secondary phloem) via active cell divisions. In most root crops, vascular cambium-driven radial growth likely determines the growth rate and yields. Recent advances in next-generation sequencing (NGS) technologies have enabled the generation of large-scale transcriptome data in developing root crops together with comprehensive genome sequence information (Firon et al., 2013; Wilson et al., 2017; Zhang et al., 2017; Machaj et al., 2018; Dong et al., 2019; Hoang et al., 2020). However, the amount and resolutions of currently available data for each root crop still leave us far from pinpointing the key processes responsible for crop yields.

With this background, in this review, to start we introduce the regulatory programs governing the development and functions of the vascular cambium in model systems, mainly Arabidopsis, and then discuss the role of the vascular cambium in the growth process and yield potential of storage roots. Second, recent advances in gene expression studies of major root crops are surveyed. These include the cassava (*Manihot esculenta*), sweet potato (*Ipomoea batatas*), carrot (*Daucus carota*), sugar beet (*Beta vulgaris*), and radish (*Raphanus sativus*). Third, based on the notion that the growth of the surveyed root crops is wholly driven in the presence of an established vascular cambium, we present our genome-wide cross-species analysis of recently published transcriptome data from representative root crops, as well as high-resolution cell type-specific expression data from Arabidopsis roots. This effort led to the identification of genes involved in the secondary root growth across root crops, which are highly connected in terms of their expression patterns and functions. Lastly, we suggest future engineering schemes for the improvement of root crop yields via the possible manipulation of key nodes in GRNs.

## Molecular Mechanisms Underlying Secondary Growth

Recent genome-wide studies have expanded our knowledge of secondary growth in Arabidopsis and other woody species. There are many recent reviews of the molecular mechanisms of secondary growth (Nieminen et al., 2015; Ragni and Greb, 2018; Fischer et al., 2019; Wang, 2020). Thus, here we limit the survey of the molecular mechanisms underlying secondary growth to those that are relevant to the development of storage roots.

### The Vascular Cambium Is a Key Place for Secondary Growth

Vascular plants grow continuously in apical directions (primary growth) and lateral directions (secondary growth) throughout their lifespans. This indeterminate growth is possible because meristems, established and maintained at distinct locations, protect undifferentiated stem cells as reservoirs of cells supplied for either primary or secondary growth (Cano-Delgado et al., 2010). While primary growth mainly makes shoots and roots longer, secondary growth increases the girth of the stems and roots. Primary growth is promoted by primary meristems, which are established during embryogenesis at the shoot and root apices. In contrast, secondary growth is driven by secondary meristems that are established during the post-embryonic developmental process. Secondary meristems include the vascular cambium and cork cambium, of which the vascular cambium is a primary contributor to increases in the girth (Nieminen et al., 2015).

**FIGURE 1:**
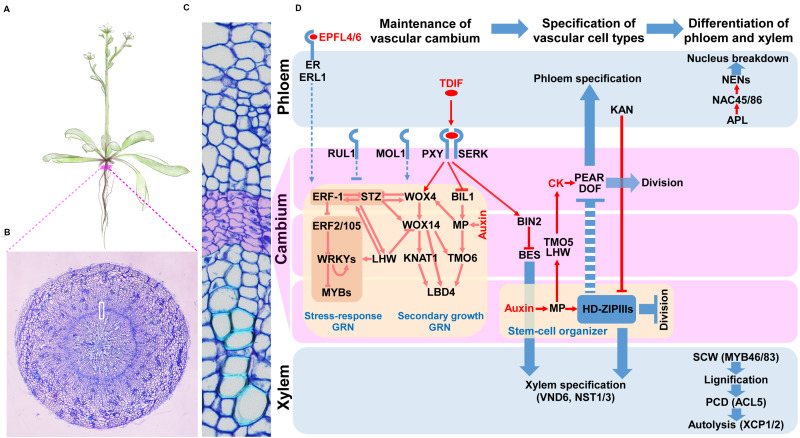
Key signaling pathways and gene regulatory networks governing the vascular cambium in Arabidopsis. **(A)** A drawing depicting Arabidopsis plant. Red bar points to root cross-section shown in **(B)**. The white box in B indicates the enlarged root cross-section representing the vascular cambium and neighboring tissues shown in **(C)**. Cambium cell layer is highlighted in purple. **(D)** Key regulators functioning during the establishment and maintenance of vascular cambium, specification of vascular cell types and differentiation of vascular cells. Pointed arrows represent activation, while blunt arrows represent inhibition. Dashed lines denote indirect regulation. Red color represents regulation of gene or protein, while blue color denotes regulation of process. Purple boxes represent cambium, while upper and lower blue boxes represent phloem and xylem precursors, respectively, as well as their derivatives. Shaded rectangles indicate co-regulation.

Secondary growth consists of three phases: (1) the establishment and maintenance of the vascular cambium, (2) the specification of vascular cell types, and (3) the differentiation of the phloem and xylem (Figure 1) (Zhang et al., 2011; Ruonala et al., 2017). How is the vascular cambium established? During primary growth, the xylem and phloem are bi-laterally established using the procambium as a border. Once the xylem and phloem during the primary growth are differentiated, cells in the procambium and its neighbors undergo a series of periclinal cell divisions, in which the division plane is parallel to the circumference of a stem or a root, giving rise to the vascular cambium in a circular form (Baum et al., 2002). The asymmetric cell divisions of vascular cambium cells add precursor cells of the secondary phloem to the outside and of the secondary xylem to the inside of the vascular cambium. These together with anticlinal cell divisions, which add more cells with an increase in the circumference, contribute to the radial growth of stems and roots (Chaffey et al., 2002). After cell type specification, cells in the secondary phloem and secondary xylem undergo differentiation. Cells in the secondary phloem differentiate into sieve-elements, companion cells, phloem fiber, or phloem parenchyma cells; while secondary xylem cells differentiate into xylem vessels, xylem fibers, or xylem parenchyma cells (Nieminen et al., 2015).

### Establishment and Maintenance of Vascular Cambium

The most commonly studied signal inputs underlying the establishment and maintenance of the vascular cambium are two peptide-receptor signaling modules, known as TDIF-PXY and EPFL4/6-ER/ERL1 modules. In the TDIF-PXY module, *CLAVATA 3* (*CLV3*)*/EMBRYO SURROUNDING REGION-RELATED 41* (*CLE41*) and *CLE44* genes are expressed in the phloem tissue, and their proteins are proteolytically processed and further modified into a peptide ligand with a length of 12 amino acids, TRACHEARY ELEMENT DIFFERENTIATION INHIBITORY FACTOR (TDIF) (Ito et al., 2006; Hirakawa et al., 2008; Ohyama et al., 2008). TDIF is released from phloem tissue, diffuses toward cambial cells, and binds to the plasma membrane-associated leucine-rich repeat receptor-like kinase (LRR-RLK) protein, PHLOEM INTERCALATED WITH XYLEM (PXY)/TDIF RECEPTOR (TDR), and its co-receptors, SOMATIC EMBRYOGENESIS RECEPTOR KINASEs (SERKs). This interaction promotes cell divisions in the cambium, inhibits xylem differentiation, and controls vascular patterning (Fisher and Turner, 2007; Hirakawa et al., 2008; Whitford et al., 2008; Etchells and Turner, 2010; Zhang et al., 2016). In the EPFL4/6-ER/ERL1 module, another LRR-receptor kinase, ERECTA (ER), and its homologs, ER-LIKE 1 (ERL1) and ERL2, regulate cambial activity. During this regulation process, ER and ERLs, expressed in phloem cells, bind to their ligands EPIDERMAL PATTERNING FACTOR-LIKE 4 (EPFL4) and EPFL6, which are produced from the epidermis (Abrash et al., 2011; Uchida et al., 2012; Uchida and Tasaka, 2013; Wang et al., 2019). Other LRR-RLKs, also known to regulate cambial proliferation in the vascular cambium, are MORE LATERAL GROWTH 1 (MOL1) and REDUCED IN LATERAL GROWTH 1 (RUL1). MOL1 and RUL1 repress and promote cambial activity, respectively; however, their ligands have not been identified (Agusti et al., 2011; Gursanscky et al., 2016).

Signal inputs from peptide-receptor interactions are integrated with GRNs that control the cambial activity. Recently, TFs and their networks controlling vascular cambium development were identified in Arabidopsis (Zhang et al., 2019; Smit et al., 2020). Starting with cambium cell-specific transcript profiling, Zhang et al. (2019) extracted more than 1,200 cambium-enriched genes from modules classified based on 23 cell type-specific datasets in the Arabidopsis root. A total of 32 cambium TFs were characterized, and 13 among them were further analyzed to uncover their roles in cambial activities. In this analysis, WUSCHEL-RELATED HOMEOBOX 4 (WOX4), WOX14, ANAC015, KNOTTED-1-LIKE 1/BREVIPEDICELLUS (KNAT1/BP), LOB DOMAIN-CONTAINING PROTEIN 3 (LBD3), and LBD4 were identified as positive regulators of cambial activities, and SHORT VEGETATIVE PHASE (SVP), RESPONSE TO ABA AND SALT 1 (RAS1), PETAL LOSS (PTL) and MYB87 were found as negative regulators. Smit et al. (2020) identified TF-promoter interactions by means of enhanced yeast one-hybrid (eY1H) screening between 812 TFs and promoters of genes regulated by TDIF-PXY. The inferred network was composed of 312 nodes and 690 edges, and its TF nodes were overrepresented by the *AP2/ERF* family TFs which are involved in stress responses (Etchells et al., 2012; Taylor-Teeples et al., 2015; Tsuda and Somssich, 2015). In this network, the authors found a feedforward loop consisting of *WOX14*, *TARGET OF MONOPTEROS 6* (*TMO6*), and *LBD4*. WOX4 and WOX14 are homeodomain TFs downstream of the TDIF-PXY module that positively regulate cambial activity (Hirakawa et al., 2010; Etchells et al., 2013). LBD4 regulates cambial cell proliferation on the phloem side in response to either WOX14 or TMO6, a Dof TF directly downstream of MONOPTEROS (MP) (Schlereth et al., 2010). Though initially found in the transcript profiling of radish cambia, multiple stress-responsive TFs, including ERF-1, ERF2, STZ, and several WRKY, as well as MYB TFs, are integrated into the GRN for cambium development (Hoang et al., 2020).

Recently, Smetana et al. (2019) found that the xylem side of the vascular cambium functions as a stem-cell organizer. HD-ZIP IIIs maintain the stem-cell organizer in a non-dividing state and promote stem-cell activity in the adjacent vascular cambium cells as well as phloem development on the other side of the vascular cambium in a non-cell autonomous manner (Brackmann et al., 2018; Smetana et al., 2019).

### Specification of Vascular Cell Types

Cells derived from asymmetric cell divisions in the vascular cambium are specified into the cell types constituting the secondary phloem to the outside and those of the secondary xylem to the inside of the vascular cambium along the radial axis. This radial patterning is established by antagonistic regulation between HD ZIP-III and KANADI (KAN) TFs (Kerstetter et al., 2001; Eshed et al., 2004; Prigge et al., 2005; Ilegems et al., 2010). HD-ZIP IIIs are expressed on the xylem side of the vascular cambium and developing xylem while KANs are expressed on the developing phloem. To establish this expression pattern, HD-ZIP III mRNAs are post-transcriptionally degraded on the phloem side by miRNAs 165 and 166 (miR165/166), as reviewed in Ramachandran et al. (2016).

A recent study introduces other players in this process, PHLOEM EARLY DOF 1 (PEAR1) and its homologs (Miyashima et al., 2019). PEARs (i.e., PEAR1 and its homologs) regulate cell divisions and specifications for phloem cell types in the vascular cambium. PEAR1, for example, is produced in the phloem precursor cell but can move to the xylem side to control the expression of HD-ZIP III TFs. HD-ZIP IIIs in turn suppress the expression of PEARs, thereby balancing xylem and phloem development.

### Differentiation of the Phloem and Xylem

The xylem vessel, a representative cell type in xylem tissue, differentiates through the following steps: secondary cell-wall (SCW) deposition, lignification, programmed cell death (PCD), and autolysis [see Escamez and Tuominen (2014) for a review]. Several NAC-domain TFs, i.e., VASCULAR RELATED NAC DOMAIN TFs (VNDs), have been identified as master regulators of xylem vessel differentiation. *VND7* and *VND6* induce protoxylem and metaxylem differentiation, respectively. Together with NAC SECONDARY WALL THICKENING PROMOTING FACTOR 1 (NST1), SECONDARY WALL-ASSOCIATED NAC DOMAIN PROTEIN 1 (SND1)/NST3 promotes xylem fiber differentiation, as reviewed in Zhang et al. (2011). These NAC TFs activate another set of TFs that regulate downstream processes. Among them, several MYB TFs, thermospermine signaling, and XYLEM CYSTEINE PEPTIDASE 1 (XCP1) and XCP2 play important roles in the SCW synthesis, PCD, and autolysis processes, respectively, as reviewed in Nieminen et al. (2015). Importantly, using an eY1H screen with a subsequent network analysis, Taylor-Teeples et al. (2015) reported the GRN that regulates SCW synthesis.

Sieve elements and companion cells are major cell types of phloem tissue. ALTERED PHLOEM DEVELOPMENT (APL), a MYB TF-encoding gene, was the first identified gene regulating phloem cell differentiation (Bonke et al., 2003), and its downstream TFs, NAC45 and NAC86, promote the expression of NAC-DEPENDENT EXONUCLEASEs (NENs), which mediate the breakdown of the nucleus during sieve element differentiation (Furuta et al., 2014).

### Integration of Hormone Signaling in the Vascular Cambium

Hormones are very important signal inputs that establish and maintain the vascular cambium. For details about these processes, see reviews by Zhang et al. (2011), Nieminen et al. (2015), Ragni and Greb (2018), Fischer et al. (2019), and Wang (2020). Here, we highlight recent findings very briefly.

Auxin plays a critical role in maintaining stem-cells in the vascular cambium. Auxin activates MP in the xylem precursors, after which, MP binds to the *WOX4* promoter to activate *WOX4* transcription, thereby promoting cambial activity (Brackmann et al., 2018; Han et al., 2018). MP in the xylem precursor also promotes the expression of *ATHB8*, one of the HD-ZIP IIIs, which maintains a stem-cell organizer, as described by Smetana et al. (2019).

TMO6, a direct target of MP, and other Dof TFs including PEARs, are regulated by cytokinins. The expression of these TFs increases with an increase in the cytokinin level, leading to higher levels of cambial activity (Miyashima et al., 2019; Smet et al., 2019). TMO5 and LONESOME HIGHWAY (LHW), bHLH TFs, function as key factors turning on TMO6 and DOF2.1 by forming a TF complex that promotes the production of cytokinins (De Rybel et al., 2014; Ohashi-Ito et al., 2014).

Additionally, brassinosteroid (BR) components are integrated into vascular cambium signaling. BRI1-EMS SUPRESSOR 1 (BES1), a TF executing BR signaling module, promotes xylem differentiation. TDIF-PXY signaling suppresses xylem differentiation by upregulating BRASSINOSTEROID-INSENSITIVE 2 (BIN2), which represses BES1 via phosphorylation (Kondo et al., 2015).

## Storage Root Formation as an Example of Secondary Growth Driven by the Vascular Cambium

### Origin and Anatomy of Storage Roots in Major Root Crops

Storage organs in root crops differ from those in tuber crops (e.g., potato) in that root crops derive from roots, whereas tuber crops derive from underground stems. Storage roots develop via the thickening of the primary or adventitious roots. Amongst root crops, cassava, and sweet potato are propagated vegetatively, whereas carrot, sugar beet, and radish are seed-propagated (Villordon et al., 2014). Typically, the first group develops storage roots from several adventitious roots, whereas the second develops these types of roots from an embryonically derived primary root. Therefore, despite the fact that secondary growth and root thickening in these major root crops are driven by the same stem-cell type, they exhibit certain differences with regard to how root thickening is initiated and in the number of storage roots per individual.

In cassava, active secondary growth occurs in selected adventitious roots (El-Sharkawy, 2004). In sweet potato, similar to cassava, root thickening begins rapidly in some adventitious roots as a result of active cell divisions in the vascular cambium toward the xylem side (Wilson and Lowe, 1973). Adventitious roots contain mostly xylem fibers and vessels, whereas storage roots contain mostly xylem parenchyma cells that store starch (Chaweewan and Taylor, 2015; Siebers et al., 2017). Typically, in cassava and sweet potato, multiple storage roots develop per plant.

**FIGURE 2:**
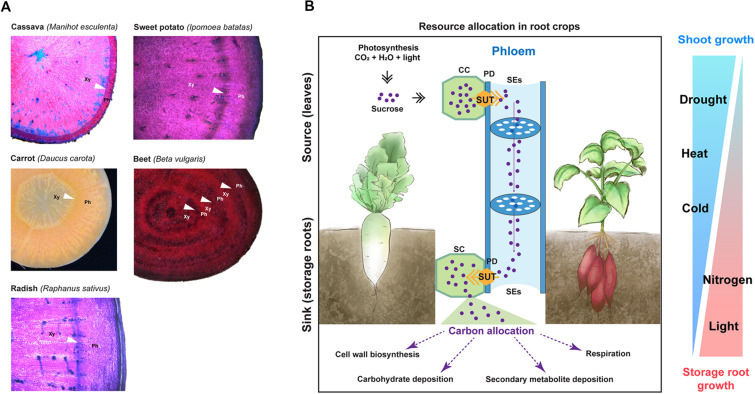
Anatomy of storage roots and environmental factors influencing storage root development. **(A)** Root cross-sections of major root crops including cassava, sweet potato, carrot, sugar beet, and radish. Cross-sections of cassava, sweet potato, and radish were stained with toluidine blue. Images for sugar beet and cassava were adapted from Barba-Espin et al. (2018) and Mehdi et al. (2019), respectively, which are distributed under the Creative Commons Attribution 4.0 International Licenses (https://creativecommons.org/licenses/by/4.0/). Arrow heads point to cambia. Xy, xylem side; and Ph, phloem side. **(B)** A simplified scheme depicting resource relocation from source (leaves) to sink (roots); and environmental factors influencing storage root formation and biomass in root crops. SUT, sugar transporter; CC, companion cell; PD, plasmodesmata; SE, sieve element; and SC, sink cell. On the right panel, the width of each triangle represents the influencing level of environmental factors on shoot growth (blue color) and storage root growth (red color).

Carrot, sugar beet and radish share a similar feature in their storage root formation. Their storage roots develop via thickening of the primary (tap) root. Radish storage root is formed by the thickening of the hypocotyl and upper root, which results from the activity of the vascular cambium (Usuda et al., 1999; Zaki et al., 2012). Anatomy representations and cross-sections of major root crops are illustrated in Figure 2A.

### Carbon Partitioning in Root Crops, From Source to Sink

In general, storage roots are considered to have a strong sink capacity (Bhattacharya et al., 1985; Rao and Terry, 1989; Usuda et al., 1999; El-Sharkawy, 2004; De Souza et al., 2017; Hennion et al., 2019). For their growth, photosynthates produced in the source (i.e., leaves) are translocated to the sink (i.e., roots) (Figure 2B). During this process, sucrose, a major form of translocated photosynthates, is loaded to the sieve elements from the companion cells in the source and then moves through the strands of the sieve elements toward the sink. Membrane-localized H^+^− sucrose transporters (commonly termed SUT or SUC proteins for SUCROSE TRANSPORTER) mediate the sucrose loading (Sauer, 2007). Sucrose unloading to the sink occurs via either the concentration-dependent diffusion of sucrose through the plasmodesmata or the SUT-mediated transport (Sauer, 2007). To enhance their sink strength, storage roots convert sucrose to other forms of carbohydrates, metabolites, and cell-wall components.

### Environmental and Internal Signals That Induce Storage Root Formation

In root crops, numerous factors have been reported to affect storage root formation, such as temperature (Gajanayake et al., 2014), water deficiency (Solis et al., 2014; Daryanto et al., 2016), nutrient deficiency (Villagarcia et al., 1998; Villordon et al., 2013), and hormones (Radin and Loomis, 1971; Jang et al., 2015).

Storage root formation has been reported to be significantly affected by temperature increases or decreases. For example, Gajanayake et al. (2014) reported that 26.5°C was the optimum temperature for storage root formation in sweet potato, and an increase in the temperature significantly reduced storage root formation. Both air and soil temperatures are important for storage root development, as both regulate the competition between shoot and storage root growth (Ravi et al., 2009; Gajanayake et al., 2013).

Drought affects storage root formation in root crops in that the soil moisture is critical for storage root initiation, and a lack of soil moisture irreparably alters root development. For example, Solis et al. (2014) showed that withholding water for 5 and 10 days significantly reduced storage root initiation in sweet potatoes grown in a glasshouse when compared to control plants. The authors further revealed that drought conditions during the storage root formation influenced root development and the expression patterns of stress-responsive genes and genes related to storage root formation in sweet potatoes. It is important to understand that the extent of the impact varies and depends on the phenological phase during which the drought occurs. However, all root crops are particularly sensitive to drought during the root thickening period (Daryanto et al., 2016).

In addition, in sweet potato, root branching and the initiation of storage root formation are affected by the availability of nitrogen (Villagarcia et al., 1998; Villordon et al., 2013). For example, the expression of *ARABIDOPSIS NITRATE REGULATED 1* (*ANR1*), known to regulate lateral root development in response to nitrate (Zhang and Forde, 1998), was found to be induced in roots generating storage roots in sweet potatoes (Firon et al., 2013).

Besides environmental cues, hormones are also considered as a factor driving storage root formation. The involvement of cytokinins and abscisic acid (ABA) during storage root formation process has been well reported (Matsuo et al., 1983, 1988; Suye et al., 1983; Sugiyama and Hashizume, 1989; Nakatani and Komeichi, 1991a, b; Lebedeva et al., 2015). In sweet potatoes, *IbMADS1* (*I. batatas MADS-box 1*), a gene induced by cytokinin, functions as an important integrator of hormone networks for storage root formation (Ku et al., 2008). Several other studies have shown that the internal balance between ABA and cytokinins is essential for storage root development (Matsuo et al., 1983; Wang et al., 2005). Apart from crosstalk with ABA, cytokinin should function in balance with auxin (Lebedeva et al., 2015). For the radish, Lebedeva et al. (2015) found that an altered cytokinin-auxin balance resulted in tumor formation on radish roots, involving abnormal cambial activity.

### Environmental and Internal Signals Influencing the Storage Root Biomass

In root crops, environmental factors such as temperature, light, drought, and hormones have been reported to affect the storage root biomass. For example, radish reportedly shows increases in storage root growth and photosynthesis at low temperatures via enhanced sink strength of the storage root (Sirtautas et al., 2011).

Light is a crucial factor that significantly affects the storage root biomass. In root crops, the light intensity and photoperiod levels reportedly regulate storage root development. Zha and Liu (2018) showed that red light accompanied by an appropriate proportion of blue light promotes the growth and enlargement of the storage root in the radish. The authors further revealed that the biomass of both shoots and storage roots of radishes decrease as the light intensity decreases, but the reduction in the storage organ was more dramatic than the reduction in the shoot biomass. It was also reported that the net photosynthetic rate of plants increases with an increase in the light intensity within a specific range, while only the chlorophyll content in new leaves increased with an increase in the light intensity (Hole and Dearman, 1993; Marcelis et al., 1997; Zha and Liu, 2018). Wang Q. et al. (2014) reported that in response to weak light, sweet potato plants with reduced the storage root biomass showed decreases in the photosynthetic rate, adenosine triphosphatase (ATPase) activity, ribulose 1,5-bisphosphate carboxylase (RuBPCase) activity, and soluble sugar content. The authors suggested that the photosynthesis activity may be decreased owing to the decrease in the activity of RuBPCase, a key enzyme in photosynthesis. Furthermore, the decrease in the ATPase activity level reduced the translocation of photosynthate from the leaves to the roots, which consequently reduced the storage root biomass.

Among other environmental factors, drought is regarded as the most important factor affecting the storage root biomass. In carrots and other root crops, it was shown that a water-deficient condition reduces the capacity for the storage root biomass production (Van Heerden and Laurie, 2008; Reid and Gillespie, 2017). However, this seems to be primarily related to the reduction in photosynthesis due to stomatal closure. While comparing two varieties of sweet potato, Van Heerden and Laurie (2008) suggested that the storage root biomass is affected by reduced photosynthesis caused by stomatal closure in water-deficient conditions.

Plant hormones such as gibberellins (GAs), cytokinins, and ABA have been reported to affect the storage root biomass. GAs are crucial stimulators of plant growth; however, an imbalance in the GA endogenous concentration can reduce the storage root biomass. In the carrot, it has been reported that the exogenous treatment of GA_3_ inhibits storage root growth (Wang et al., 2015b). GA_3_ also was shown to enhance the xylem region, which may weaken the root texture and quality. The inhibition of storage root growth is attributable to changes in the xylem developmental process. On the other hand, ABA has been reported to modulate the thickening of storage roots by enhancing meristem cell division, particularly at the secondary meristem in the xylem. More recently, Jang et al. (2015) showed that cytokinin functions as a key modulator controlling storage root growth through the regulation of cambial cell proliferation. The authors found that the cytokinin response was dynamically regulated, positively correlated with the secondary growth activity, and was stronger in the root cambium compared to the adjacent tissues.

## Gene Expression Studies of Storage Root Formation, a Current Progress in Major Root Crops

Despite the economic importance of root crops, there is limited information regarding GRNs that control their storage root formation processes and yields. The majority of the information is derived from separate studies, and overall it remains fragmentary. Here, we summarize the currently available data generated in recent years on major root crops to get a glimpse of gene regulatory programs that are shared or distinctive among them.

### The Development of Storage Organs From Different Origins May Share Similar Gene Regulatory Programs

As discussed in Section “Storage Root Formation as an Example of Secondary Growth Driven by the Vascular Cambium,” multiple external and internal signals come into play to trigger storage root formation. However, most molecular genetic studies of the formation of storage organs were conducted using potato (*Solanum tuberosum*) tubers, which derive from underground stems (Sarkar, 2008; Hannapel et al., 2017). Signaling components that trigger the flesh tuber formation processes identified in these studies include the MADS-box (Rosin et al., 2003b; Sojikul et al., 2015), KNOTTED1-LIKE HOMEOBOX (KNOX)/POTATO HOMEOBOX 1 (POTH1)-like (Rosin et al., 2003a; Banerjee et al., 2006; Tanaka et al., 2008; Guo et al., 2014) BEL5-LIKE HOMEODOMAIN (BEL5) (Banerjee et al., 2006) SELF-PRUNING 6A/FLOWERING LOCUS T (SP6A/FT)-like (Navarro et al., 2011) and POLYPYRIMIDINE TRACT-BINDING PROTEIN 1/6 (PTB1/6) gene families (Cho et al., 2015).

MADS-box (e.g., potato StPOTM1, sweet potato IbMADS1) and KNOX/POTH1 TFs are known to mediate storage organ development (Rosin et al., 2003a, b; Ku et al., 2008; Tanaka et al., 2008; Sojikul et al., 2015). In the potato, StKNOX/POTH1 interacts with StBEL5 to regulate the corresponding target genes during this process (Chen et al., 2004). One interesting aspect with regard to potato tuber formation is the action of long-distance mobile signals from source to sink: *StBEL5* mRNA and its target StSP6A/FT protein move from leaves to stolons (underground stems) to promote their growth to tubers (Sharma et al., 2016; Abelenda et al., 2019). St PTBs function as RNA-binding proteins facilitating the movement of mRNA *StBEL5* and its homologs (Ghate et al., 2017). StSP6A then enhances the sink strength by suppressing the leakage of sucrose in the sink through direct interactions with StSWEET11, a sucrose efflux transporter (Sharma et al., 2016; Abelenda et al., 2019). StBEL5-POTH1 promotes genes known as potato tuber markers: *ISOPENTENYL TRANSFERASE* (*IPT*), *YUCCA 1* (*YUC1*), *AUXIN RESPONSE FACTOR 8* (*ARF8*), *AGAMOUS-LIKE 8* (*AGL8*), *GA2 oxidase* (*GA2OX1*), *GA3OX1*, *GA20OX1*, *LONELY GUY 1* (*LOG1*), *PIN-FORMED 1* (*PIN1*), *PIN2*, *PIN4*, and *CYCLING DOF FACTOR 1* (*CDF1*) (Sharma et al., 2016). Based on the evolutionary conservation of core tuber regulators in other root crops, Natarajan et al. (2019) suggest that these genes may also play pivotal roles in storage root development in root crops.

### Gene Expression Studies of Major Root Crops

Over the last decade, with the aid of NGS technologies, there is a growing amount of genome-wide expression data generated for root crops. Together with reference transcriptomes, the release of reference genomes for the cassava (Wang W. et al., 2014; Bredeson et al., 2016), sweet potato (Wu et al., 2018), carrot (Xu et al., 2014; Iorizzo et al., 2016; Wang F. et al., 2018), sugar beet (Dohm et al., 2014), and radish (Kitashiba et al., 2014; Mitsui et al., 2015; Jeong et al., 2016) has facilitated more comprehensive gene expression studies related to storage root development. These include the profiling of genes involved in, for example, the regulation of storage root formation in the cassava (Yang et al., 2011; Wilson et al., 2017); understanding storage root formation, carbohydrate metabolism and carotenoid biosynthesis in the sweet potato (Tao et al., 2012; Firon et al., 2013; Wang et al., 2015c; Dong et al., 2019); root development, hormonal control and carotenoid biosynthesis in the carrot (Wang et al., 2015a; Ma et al., 2018; Machaj et al., 2018); taproot growth and sucrose accumulation in the sugar beet (Zhang et al., 2017); and root formation and glucosinolate biosynthesis, anthocyanin synthesis, stress response, and the relationship between storage root growth and stress responses in the radish (Wang et al., 2013; Mitsui et al., 2015; Xie et al., 2015; Xu et al., 2015; Gao et al., 2019; Hoang et al., 2020).

In general, these studies resulted in several thousands of candidate genes (differentially expressed genes, DEGs) whose functions are potentially linked to the molecular basis of the formation of storage roots. By comparing whole transcriptome data from cassava fibrous roots and developing and mature storage roots, Yang et al. (2011) identified a total of 20 significantly enriched pathways during storage root development. Among these, the authors highlighted pathways such as those related to glucide metabolism, zeatin biosynthesis, lipid biosynthesis and other secondary metabolic processes as those related to starchy storage root development. More recently, Wilson et al. (2017) constructed a gene expression atlas for cassava key tissues and organs, including the root apical meristem along with fibrous and storage roots. By clustering these data, the authors uncovered inherently distinct transcript clusters with strong, constitutive and tissue/organ-specific expression patterns. For those transcripts preferentially expressed in fibrous roots and storage roots, the authors found that the gene ontology (GO) terms related to “translation,” “proteolysis,” and “intracellular” were enriched in the former; while “zinc ion” and “phosphatidylinositol binding” were enriched in the latter. Collectively, these studies suggest the involvement of several candidate TFs in storage root formation and starch accumulation in the cassava.

In the sweet potato, whole transcriptome analyses suggested the involvement of genes related to lignin biosynthesis, sucrose and starch biosynthesis during storage root formation (Tao et al., 2012; Firon et al., 2013; Wang et al., 2015c). Several well-known TFs were also identified, including *IbMADS1*, *SHORTROOT* (*SHR*), *BEL1-LIKE HOMEODOMAIN 1* (*BLH1*), *KNOX1* and *ETHYLENE RESPONSE FACTORs* (*ERFs*). Collectively, the summary by Tanaka (2016) indicated that important genes involved in sweet potato root formation are those related to carbohydrate metabolism, sugar signaling, lignin biosynthesis, cell division, development and transcription. More recently, Dong et al. (2019) analyzed the transcriptome and proteome from the fibrous roots and four developmental stages of sweet potato storage roots, finding that genes related to meristem/cambium development, starch biosynthesis and hormones were differentially expressed during storage root formation. The data also suggested several other TFs, including those related to meristem development (*LBD4*, *WOX14*, and *TMO6*) and starch biosynthesis (*NAC1*, *bZIP*, and *MYB*) in the sweet potato.

In the carrot, studies have linked genes active during carrot storage root formation to multiple metabolic, molecular, and biological processes (Wang et al., 2015a; Machaj et al., 2018). Machaj et al. (2018) identified many TFs belonging to multiple TF families, including a previously known “domestication gene” *DcAHLc1*. In the sugar beet, Zhang et al. (2017) found numerous genes important for storage root formation and sucrose accumulation. These include genes encoding TFs; genes involved in the plant production of BR, auxin, and cytokinin; and genes involved in hormone signal transduction pathways and sucrose metabolism. In the radish, Mitsui et al. (2015) reported gene clusters related to “stress and stimulus responses,” “transport,” and “membrane activities” that were enriched during early root development; while “ribosomal activity,” “structural molecule activity,” and “translation” were enriched in primary root thickening; and “membrane activities,” “transcription,” and “cell development” more enriched during secondary root thickening.

A study by Hoang et al. (2020), released at the time of the writing this review, presents high-resolution transcriptome data in a root crop for the first time. In this study, 17 tissue-specific transcriptome datasets were generated in the radish by the means of laser capture micro-dissection (LCM), covering the phloem cortex, cambium, and xylem parenchyma from storage roots at three different developmental stages (5, 7, and 9 weeks post seed planting) in two inbred lines with contrasting growth levels and yields. Using these data, the authors found that the expression levels of the key cambium regulators and hormone signaling components (discussed in section “Molecular Mechanisms Underlying Secondary Growth”) are well conserved and enriched in the radish cambium. A comparative transcriptome analysis against Arabidopsis root data (Zhang et al., 2019) revealed conserved GRNs. Among these, the authors focused on a GRN enriched with stress-responsive TFs, which are highly expressed in the cambia of the inbred line with low yields. The authors then selected a set of cambium-enriched growth regulators and the aforementioned stress-responsive TFs, inferring the GRN through a series of perturbation experiments. The results revealed that stress-responsive TFs in the GRN closely control root secondary growth, with a highly connected hub in the identified GRN known as *ERF-1*, a stress-responsive gene. In this GRN, *ERF-1* appears to act as a balancer between secondary growth and stress responses.

### The Main Challenge of Analyzing Current Gene Expression Data of Root Crops

Collectively, the recent gene expression studies focusing on root crops have provided a valuable foundation for understanding the molecular basis of storage root development. However, most of these studies were performed using whole organs, inevitably leading to a lack of resolution regarding tissue-specific gene expression patterns despite the hundreds or thousands of candidate genes identified. Elucidating gene regulatory mechanisms is further complicated by the fact that regulatory genes (e.g., TFs) tend to be expressed at a low level or in a tissue-specific manner. This results in expression levels of regulatory genes diluted or under detection in the transcriptome data when that data originate from a whole root sample. It is also important to mention here that different expression fold-change cutoffs (e.g., 2 and 4) were used to detect DEGs in the aforementioned transcriptome studies. While this was not likely to affect highly differentially expressed genes, it could change the data interpretation of those genes expressed with marginal differences.

We thus asked whether the currently available information obtained from one root crop species has the potential to be applied to other root crops. This will facilitate the more effective utilization of currently available data to analyze the key processes involved in storage root formation and yields. To this end, we performed a cross-species meta-analysis on published expression data of major root crops. These results are presented in the next section.

## Cross-Species Comparative Analysis Identified Conserved Key Cambium Grns That Function in Root Crops

Previous works on Arabidopsis and woody plants (i.e., *Populus*) suggested that conserved regulators in the cambium control secondary growth in stems (Schrader et al., 2004; Du et al., 2009; Zhang et al., 2011; Ragni and Hardtke, 2014; Ragni and Greb, 2018). Identifying conserved gene regulatory programs would allow the application of knowledge obtained from model systems to other plants, especially those with complex genomes. Furthermore, this helps to delineate conserved programs across diverse plant species from species-specific regulatory programs that make the growth form of each species unique (Schrader et al., 2004; Siebers et al., 2017; Chen et al., 2019).

**FIGURE 3:**
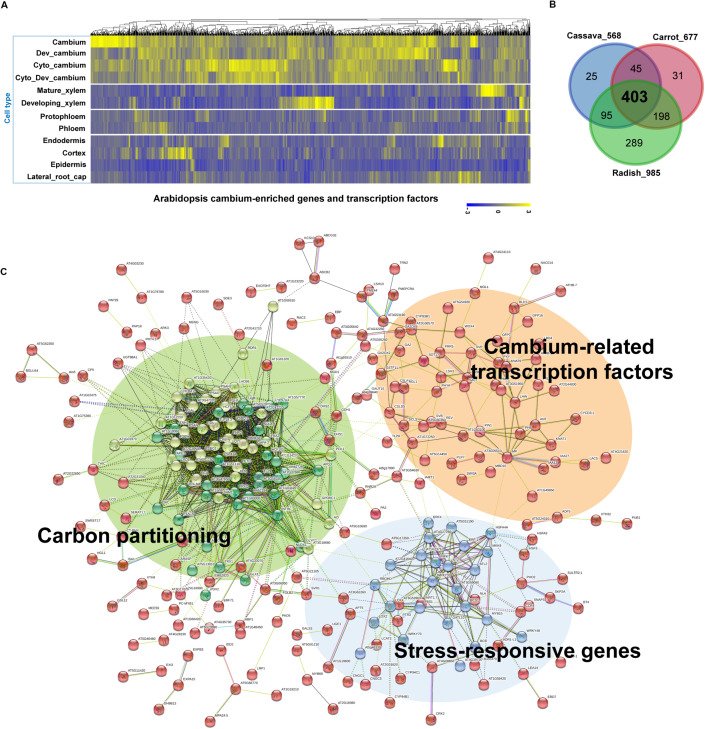
A cross-species comparison of root transcriptome data from major root crops to identify conserved networks critical for storage root growth and yields. **(A)** Expression of 1,222 cambium-enriched genes in Arabidopsis (Zhang et al., 2019), in selected cell types including cambium, developing cambium (Dev_cambium), cambium after cytokinin treatment (Cyto_cambium), developing cambium after cytokinin treatment (Cyto_ Dev_cambium), mature xylem, developing xylem, protophloem, phloem, endodermis, cortex, epidermis, and lateral root cap. Data were gene-wise normalized, clustered, and plotted using the Pheatmap R package (https://cran.r-project.org/web/packages/pheatmap/). Each vertical line represents one gene. **(B)** Identification of common DEGs among major root crops. Transcriptome data were obtained for cassava (Wilson et al., 2017), carrot (Machaj et al., 2018), and radish (Hoang et al., 2020). Data were plotted using the Venn tool (http://bioinformatics.psb.ugent.be/webtools/Venn/). **(C)** Kmeans clustered (*k* = 3) network of 403 common DEGs in panel **(B)** inferred from the STRING database version 11.0 (Szklarczyk et al., 2015). Genes (nodes) are connected by edges, based on a default setting of evidence. For visualization, the unconnected nodes were hidden in the network. A higher resolution of this panel is provided in Supplementary Figure S2.

For our cross-species meta-analysis (see Supplementary Figure S1 for a detailed workflow chart), we selected a set of 1,222 Arabidopsis (*At*) genes (Supplementary Table S1) that were reported to be enriched in the developing cambia (Zhang et al., 2019). The expression data indicated that the majority of these genes exhibit expression levels which are enriched in the developing cambia in comparison to other major cell types in the root (Figure 3A). We then matched these 1,222 cambium-enriched genes to the corresponding putative orthologs used in storage root transcriptome studies on the cassava (Wilson et al., 2017), carrot (Machaj et al., 2018), and radish (Hoang et al., 2020), and thereby identifying 1,280 orthologous genes in cassava, 1,581 genes in carrot and 2,206 genes in radish data. The orthologous gene pair annotations were obtained from Phytozome version 12.1^1^ for the cassava and from respective studies for the carrot and radish. Three transcriptome datasets were selected because these represent different storage root origin types (i.e., vegetatively propagated vs. seed-propagated) and sample collecting methods (i.e., whole root vs. micro-dissected tissues), at the same time, they are easily comparable due to the usage of the same NGS technology. For a cross-species comparison, the expression levels of the identified orthologous genes were retrieved for samples reported in each study. To identify DEGs, the expression data were then compared between storage and fibrous roots (for cassava); among young, developing and mature roots (for cultivated carrot); and between the cambium and cortex or parenchyma tissues at 5, 7, and 9 weeks (for radish). We retained only DEGs that exhibited a minimum | fold-change (or expression ratio)| ≥ 1.5, which included both up and down-regulated genes in each comparison. Selection of the fold-change threshold was done while considering that the expressions of many tissue-specific regulatory TFs could be diluted in whole root samples. This resulted in the total number of remaining DEGs being 938 cassava genes (corresponding to 568 *At* genes), 1,389 carrot genes (677 *At* genes), and 1,889 radish genes (985 *At* genes). The higher number of genes identified in the radish data likely stems from the higher number of developmental stages and from the dissected tissues and cultivars included in this dataset. It could also be that among the three species, the radish has the closest evolutionary proximity to Arabidopsis. Using the *At* orthologous genes, we found that a total of 403 genes, including 65 TF genes, are shared amongst the three root crop species (Figure 3B and Supplementary Table S2). Although these root crop data represent the transcriptome profiled at different resolutions, approximately a third of cambium-enriched genes in Arabidopsis roots are differentially expressed in all of these data. This suggests that these 403 cambium-enriched genes may play important roles in the storage root growth in these evolutionary divergent species. A functional enrichment analysis of the 403 shared DEGs through the STRING database version 11.0 (enrichment *p*-value < 1.0e-16) (Szklarczyk et al., 2015) indicated that “response to stimulus, GO:0050896,” “metabolic process, GO:0008152,” “biological regulation, GO:0065007,” and “photosynthesis, GO:0015979” were among the most significantly enriched GO terms (Supplementary Table S3). Two pathways, the photosynthesis and metabolic pathways, were found to be significantly enriched according to the KEGG metabolic database (Kanehisa and Goto, 2000).

**FIGURE 4:**
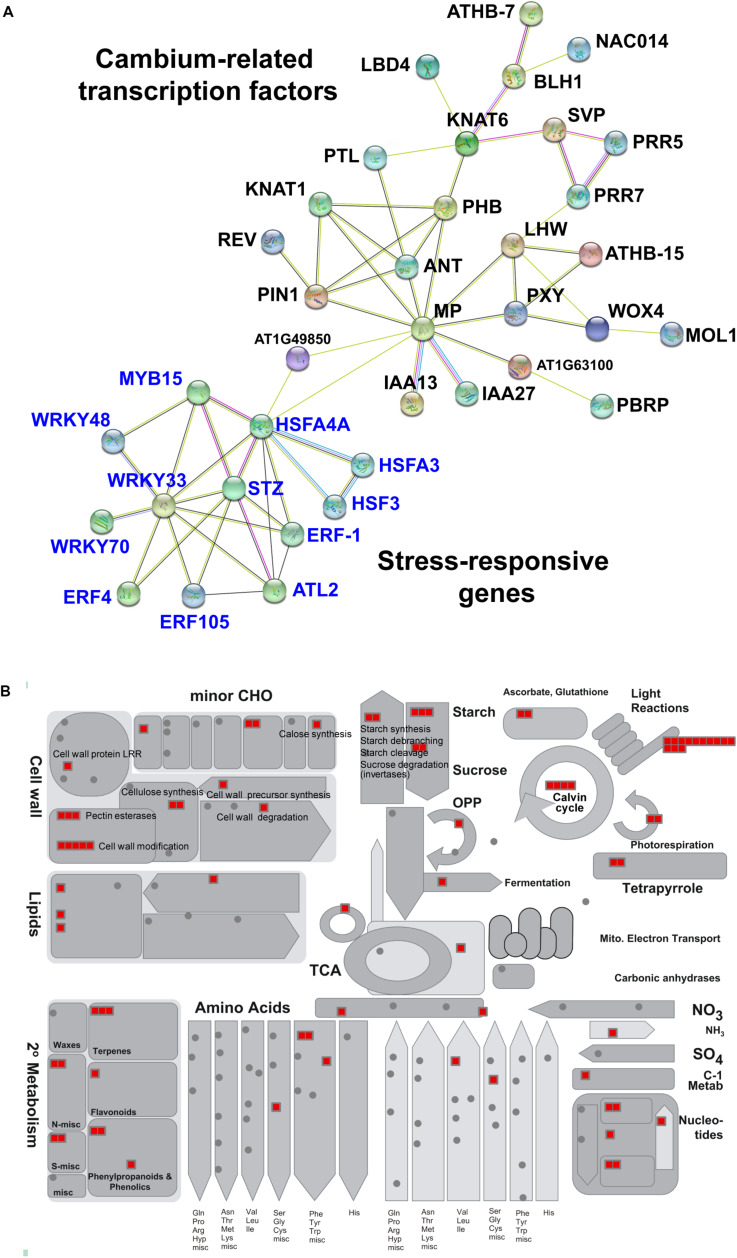
Sub-networks of cambium-related transcription factors, stress-responsive genes and genes involved in carbon partitioning in the 403 common DEGs among major root crops. **(A)** Sub-networks of 65 common transcription factors that belong to two clusters corresponding to cambium regulators and stress-responsive genes. Network was visualized by the STRING database version 11.0 (Szklarczyk et al., 2015). Genes (nodes) are connected by edges, based on a default setting of evidence. The unconnected nodes were hidden in the network. **(B)** Common genes involved in carbon partitioning analyzed by metabolism overview tool in the MapMan software version 3.5.1 (19.11.2010) (Thimm et al., 2004) and Arabidopsis TAIR Release 10 dataset. Each red block represents one gene. CHO, carbohydrate.

Interestingly, when we analyzed interaction networks for the 403 common DEGs using the STRING database (default settings, using all evidence available), we found three distinct sub-networks that are interconnected (Figure 3C and Supplementary Table S4). The first sub-network includes TFs and other regulators that are known to regulate secondary growth. These include regulators important for the establishment and maintenance of the vascular cambium (i.e., PIN1, ANT, PXY, MOL1, WOX4, KNAT1/BP, MP, LHW, PTL, SVP, and LBD4) and for xylem specification (i.e., PHB, REV, and CNA), whose functions were discussed in Section “Molecular Mechanisms Underlying Secondary Growth.” In this sub-network, we also found BLH1 (ortholog of StBEL5) and KNAT6 (ortholog of StPOTH1), which are involved in the development of potato tubers, as discussed in Section “The Development of Storage Organs From Different Origins May Share Similar Gene Regulatory Programs.” The second sub-network was consisted of stress-responsive genes, i.e., ERF-1, ERF4, ERF105, STZ, MYB15, WRKY33, WRKY48, WRKY70, ATL2, and HSFs. Our results confirmed the involvement of stress-responsive genes in root secondary growth through the formation of an interconnected GRN with growth regulators (Figure 4A). This is in line with a previous report on the radish (Hoang et al., 2020), in which a growth-stress response GRN was found to be responsible for the differences between two radish lines with contrasting growth rates. The interactions of many of these stress-responsive genes (including ERF-1, STZ, ERF105, MYB15, WRKY18, and WRKY33) with major cambium regulators (i.e., WOX4, WOX14, PXY, and ASL9) are suggested by expression data, mutant phenotypes (Hoang et al., 2020) and DAP-Seq data (O’Malley et al., 2016). This indicates that the coordination of growth-related and stress-responsive sub-networks is likely to be critical for storage root development. It was also found that these two sub-networks interacted with another gene cluster (containing the highest number of genes) related to carbon metabolism (partitioning), likely linked to the source-sink strength. MapMan (Thimm et al., 2004) revealed that genes in this tightly connected sub-network are related to carbon metabolism, in particular photosynthesis (light reactions, photosystem I, II, Calvin cycle, chloroplast and photorespiration), cell-wall biosynthesis (cellulose synthesis and cell-wall modification), and sucrose-starch metabolism (callose, sucrose, and starch) (Figure 4B).

Our meta-analysis identified genes and their potential interactions in the vascular cambium that may commonly contribute to storage root growth in root crops. These include key secondary growth regulators functionally identified in Arabidopsis, such as ANT, PXY, MOL1, WOX4, KNAT1/BP, MP, LHW, PTL, SVP, and LBD4. This indicates that storage root growth in these representative root crops is under the control of conserved vascular cambium regulators. In addition to these regulators, stress-responsive genes and carbon metabolism genes enriched in the cambium also change their expression dynamics during storage root growth. These coordinated regulations and interactions among the three gene cascades have not been reported before and therefore require further functional investigations to understand the significance of these interactions.

This meta-analysis, which started with 1,222 cambium-enriched genes derived from Arabidopsis, helps to identify novel regulatory programs driving storage root growth. However, it still misses interesting regulation aspects because Arabidopsis does not produce storage roots. As an example, only two genes, *BLH1* (*StBEL5*) and *KNAT6* (*StPOTH1*), out of four tuber regulators were included in the list of cambium-enriched genes. However, upon an investigation of available expression data, we found that putative orthologs of *StPTB1/6*, which assist in the movement of *StBEL5* in the potato, are also differentially expressed in three major root crop species, with enrichment in the radish cambia. The expression of orthologs of *StSP6A*/*FT*-like, on the other hand, was not detected in the cassava and carrot data; but generally low (i.e., FPKM <3) in the radish data, with enrichment in 7- and 9-week cambia.

## Modifying the Cambium-Driven Secondary Growth Network and Potential Improvement of Root Crop Yields

One major application of our understanding of the molecular mechanisms underlying secondary growth in storage roots is to engineer root crops with increased yields and growth rates and better stress resistance. Based on current knowledge of GRNs underlying storage root formation in major root crops, with the integration of hormone signaling pathways and environmental factors, we discuss two potential engineering strategies for improving root crop yields.

First of all, improved growth and yields of root crops could be achieved by altering the nodes with a large impact on the cambium GRNs. At least two different approaches could be taken to define nodes with high impacts: one is to define a regulatory motif with a major influence and the other is to find nodes with more connections than others in the GRN. In general, a GRN is started when a signal (i.e., developmental or environmental) is sensed by a receptor (original node), which amplifies a signaling cascade with downstream TFs in a hierarchical order (Van den Broeck et al., 2017). To regulate the downstream pathways developmentally, several feed-forward loops operate with negative feedback regulation, during which the outputs negatively regulate the original nodes to suppress certain pathways when they are not needed (Ma et al., 2009; Van den Broeck et al., 2017). Another type of feed-forward loop regulation, specifically the incoherent type, was found to enable plants to cope with environmental stresses. In this type of loop, one pathway can activate the output node, and after a delay, another pathway can then inhibit and return the output node to its original state (Ma et al., 2009; Van den Broeck et al., 2017). The identification of such regulatory motifs in the GRNs of interest would potentially suggest targets for modifications that would lead to the achievement of the desired traits.

A recent work by Zhang et al. (2019) is a good example demonstrating the usefulness of a comprehensive GRN in the vascular cambium for then engineering of secondary growth. Based on comprehensive cell type-specific transcriptome data, 32 cambium-enriched TFs were identified and the TFs affecting secondary growth were then selected. The transcriptional regulatory network inferred from gene expression changes in individual perturbations of these cambium TFs indicated *WOX4* and *KNAT1/BP* as positive regulators with the strongest influence on secondary growth in Arabidopsis. Overexpression of *WOX4* in combination with the knocking out of an inhibitory node, *PTL*, resulted in further enhancements of the cambial activity, secondary growth and biomass yields.

Second, environmental factors and signals could be incorporated as part of the cambium-driven growth GRN modifying scheme. In most cases, the phenotype is a result of the interactions between the genetic makeup and environmental conditions (Van den Broeck et al., 2017). As discussed elsewhere, secondary growth is highly responsive to environmental factors. Knowledge of a GRN integrating these two processes will help to design strategies that minimize the trade-off between growth and stress resistance. In a recent study, Kudo et al. (2019) employed a promising gene-stacking approach to manipulate the expressions of both growth regulators and stress-responsive genes in an attempt to improve plant yields and defense capabilities. The authors co-overexpressed *DREB1A* (a stress-responsive gene, whose expression is induced under drought condition) and two growth regulators, *GA5* (*GA REQUIRING 5*) and *PIF4* (*PHYTOCHROME-INTERACTING FACTOR 4*), whose expressions are suppressed under drought conditions. *GA5* is a gene from the GA biosynthesis pathway, while *PIF4* is known as a growth-improvement TF. Overall, their results suggested that, compared to *DREB1A* overexpressed plants, the double-overexpressors *GA5 DREB1A* and *PIF4 DREB1A* showed enhanced biomass accumulation and floral induction. Among these two double-overexpression lines, *GA5 DREB1A* exhibited both high stress tolerance and increased growth compared to single *DREB1A* overexpression line, indicating that this line overcame the inherent growth-stress resistance trade-off. Miller et al. (2015) found that in Arabidopsis hybrids, stress-responsive genes are normally repressed under normal conditions but are upregulated under stress conditions depending on the time of day. The downregulation of some stress-responsive genes was shown to promote growth. Hoang et al. (2020) constructed a cambium GRN consisting of selected growth-related TFs and stress-responsive TFs in Arabidopsis roots, finding *ERF-1* as a key node balancing stress responses and growth. Taken together, these findings suggest that it is possible to develop an integrated genetic strategy that affects the behavior of key growth regulators, stress-responsive genes, and signaling components of storage root formation for better or sustainable yields.

## Conclusion and Future Prospects

On-going climate change, especially the increases in temperature, is expected to affect the yields of major crops adversely (Zhao et al., 2017). As a result, unpredictable future scenarios should be incorporated into breeding programs. Root crops such as cassava and sweet potato could become food security crops for a significant fraction of the world population, especially in environmentally challenged areas. Root crop yields are mainly driven by the vascular cambium, a tissue that is sensitive to external environmental changes. Cambial activity is controlled not only by developmental regulators but also by genes that sense and respond to environmental challenges. As demonstrated in our cross-species comparison of transcriptome data during storage root growth with cambium-enriched genes in the Arabidopsis root, cambium-enriched genes are integral parts of storage root growth due to the formation of a highly wired GRN that coordinates secondary growth in response to environmental cues.

To engineer root crops with high yields and good stress resistance, a clear understanding of the detailed GRN is critical. The meta-analysis based conserved GRN discussed in this review could serve as a useful starting platform in this context. However, knowledge mainly relying on Arabidopsis has an inherent limitation given that Arabidopsis plants do not produce storage roots. The establishment of a model system for root crops, therefore, will likely benefit the research community by allowing studies of certain unique aspects of storage root-bearing crops. In such a case, besides the potato tuber, the radish could serve such a purpose given its several advantages over other root crops, as per the discussion in Hoang et al. (2020).

## Author Contributions

J-YL and NH conceptualized and designed the study. NH performed the cross-species analysis and prepared the first draft. CP contributed to section “Molecular Mechanisms Underlying Secondary Growth.” MK contributed to the writing of section “Storage Root Formation as an Example of Secondary Growth Driven by the Vascular Cambium,” and partially to the concept of Figure 2. J-YL supervised, interpreted the analysis, and revised the draft. All authors read, discussed, edited, and approved the final manuscript.

## Conflict of Interest

The authors declare that the research was conducted in the absence of any commercial or financial relationships that could be construed as a potential conflict of interest.

## Footnotes

1 https://phytozome.jgi.doe.gov

## Supplementary Material

FIGURE S1 A workflow chart showing main steps used in the meta-analysis of cross-species transcriptome data. Arabidopsis data were obtained from Zhang et al. (2019). Cassava data were downloaded from http://shiny.danforthcenter.org/cassava_atlas/ as specified in Wilson et al. (2017). Carrot data were obtained from Machaj et al. (2018). Radish data were obtained from Hoang et al. (2020). Gene–gene interactions were analyzed by the STRING database (Szklarczyk et al., 2015) version 11.0, accessed on 15th February 2020. Functional analyses including the GO enrichment and KEGG pathways were done through the STRING data tools. Overview metabolic pathway analysis was done using the MapMan software version 3.5.1 (19.11.2010) (Thimm et al., 2004) and Arabidopsis TAIR Release 10 dataset. TF, transcription factors.

FIGURE S2 A high-resolution of Figure 3C showing gene–gene interactions among 403 common DEGs identified in the selected root crops datasets, with detailed gene names. The figure was generated by the STRING database version 11.0, accessed on 15th February 2020.

TABLE S1 List of 1,222 cambium-enriched genes identified in Arabidopsis root data.

TABLE S2 Lists of DEGs, 403 common DEGs and 65 common TFs identified among the three major root crops, the cassava, carrot and radish.

TABLE S3 Gene ontology (GO) term functional analysis of 403 common DEGs analyzed by the STRING database.

TABLE S4 Gene network interactions of 403 common DEGs analyzed by the STRING database.
