# Supplementary figures and images for: Gene Regulatory Network Guided Investigations and Engineering of Storage Root Development in Root Crops
Source: Front Plant Sci. 2020 Jun 17;11:762. doi: 10.3389/fpls.2020.00762 (PMC7313660; doi:10.3389/fpls.2020.00762)

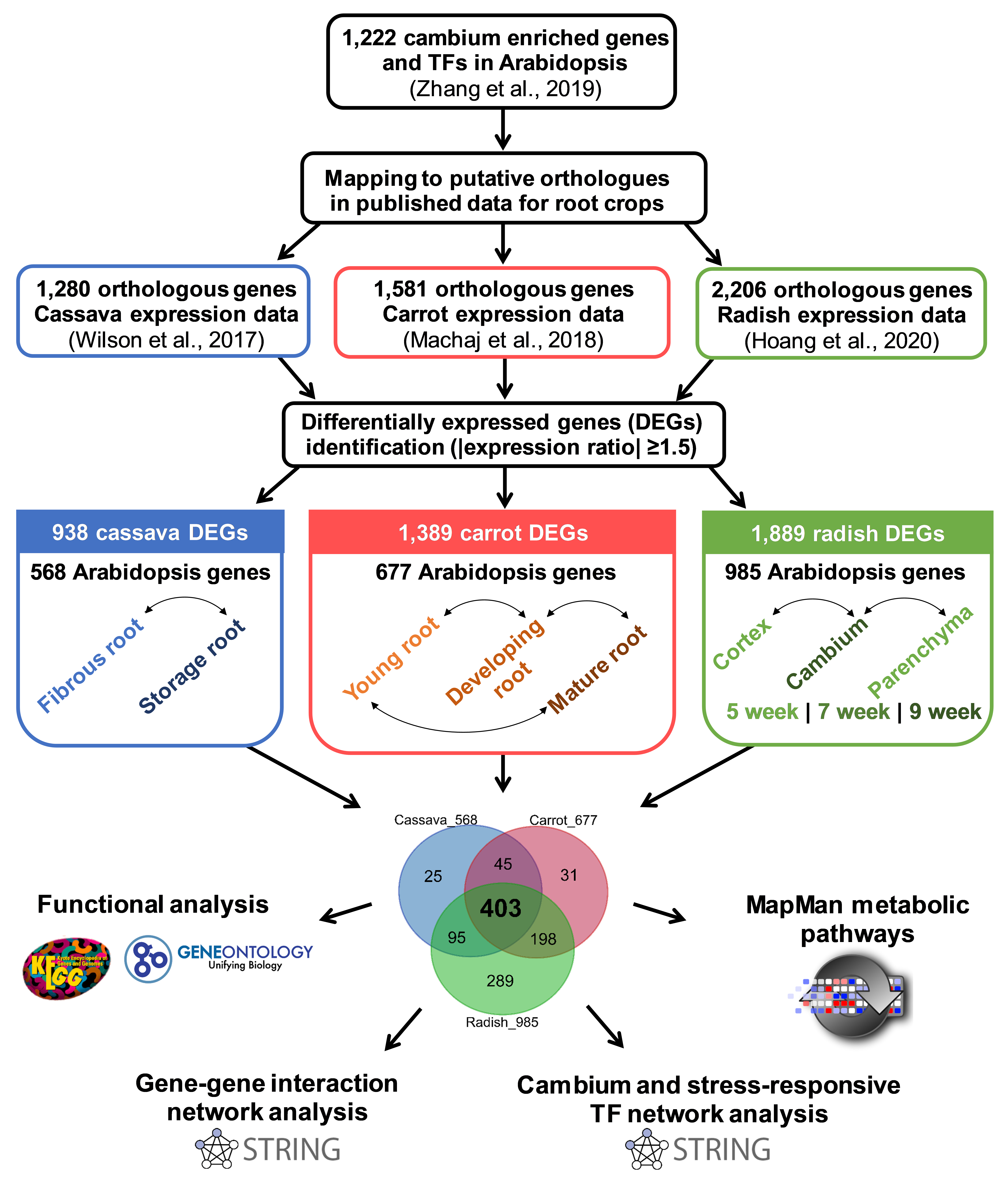

Supplement: FIGURE S1 — A workflow chart showing main steps used in the meta-analysis of cross-species transcriptome data. Arabidopsis data were obtained from Zhang et al. (2019). Cassava data were downloaded from http://shiny.danforthcenter.org/cassava_atlas/ as specified in Wilson et al. (2017). Carrot data were obtained from Machaj et al. (2018). Radish data were obtained from Hoang et al. (2020). Gene–gene interactions were analyzed by the STRING database (Szklarczyk et al., 2015) version 11.0, accessed on 15th February 2020. Functional analyses including the GO enrichment and KEGG pathways were done through the STRING data tools. Overview metabolic pathway analysis was done using the MapMan software version 3.5.1 (19.11.2010) (Thimm et al., 2004) and Arabidopsis TAIR Release 10 dataset. TF, transcription factors. [file Image_1.TIF]
